# The Power of the General Medical Council

**Published:** 1894-12-29

**Authors:** 


### EDITOR'S LETTER-BOX.

### THE POWERS OF THE GENERAL MEDICAL COUNCIL.

"Heretic" writes: It becomes a matter of grave importance to the medical profession to know where the line is to be drawn in defining "infamous conduct in a professional sense." Every year the General Medical Council narrows the path upon which the humble general practitioner may tread, and remain within the bounds of orthodoxy and in the illumination of the rays shed by the Council and from the high places of the profession.

Nobody can complain when the Council punishes men for such dishonesty as "covering," or other crooked ways; but the latest removal from the register is one which has been made for a reason which comes very near to the ground upon which a large number of medical men are treading. A medical practitioner of forty years' standing, and of honourable life, has been removed from the register because he has translated a medical work written by an Italian. The work in question contains matter with which the profession cannot agree (personally I think it absurd), but the gentleman who made the translation, although aware that the writer of the original is not a qualified medical man, yet quite honestly believes that what we see as nonsense is actually a true and practicable system of therapeutics.

This is the offence styled "infamous conduct" by the latest decision of the Council; and as the Medical Act provides that the Council is the sole judge of what constitutes infamous conduct, and that against that decision there shall be no appeal, the gentleman of whom I speak is absolutely helpless, degraded from professional status, and driven, against his will, into the ranks of the quacks.

A further aggravation of the insult is that, when the translator found that his work was hurting the feelings of the Council, he withdrew the book, and also that there is a clause in the Act providing that no name shall be removed from the register on account of adhesion to any theory of surgery or medicine.

The laity naturally conclude, when they hear that a man has been guilty of "infamous conduct," that he has committed some grossly immoral act, and social degradation is likely to follow upon the action of the Council; but where the matter becomes so dangerous is when the writing of medical books and pamphlets bringing forward the writer's particular beliefs is called infamous conduct. Considering what a large number of medical men do write monographs upon some point in which they are specially interested in medical or surgical practice, the precedent made by the Council is a very serious one. I have myself been guilty of writing a pamphlet (several, in fact), in which I advocated a particular method of surgical treatment in certain cases of deformity which I have studied, and my opinions differ considerably from those held by many of my professional brethren. I begin to fear that I must avoid in future putting on record any results of practice or opinions which run counter to the experience or opinions of anyone else, or I, too, may be found guilty of "infamous conduct."

